# A novel amplitude binning strategy to handle irregular breathing during 4DMRI acquisition: improved imaging for radiotherapy purposes

**DOI:** 10.1186/s13014-019-1279-z

**Published:** 2019-05-14

**Authors:** Z. van Kesteren, A. van der Horst, O. J. Gurney-Champion, I. Bones, D. Tekelenburg, T. Alderliesten, G. van Tienhoven, R. Klaassen, H. W. M. van Laarhoven, A. Bel

**Affiliations:** 1 Department of Radiation Oncology, Amsterdam UMC, University of Amsterdam, Meibergdreef 9, 1105AZ Amsterdam, The Netherlands; 2 Department of Radiology and Nuclear Medicine, Amsterdam UMC, University of Amsterdam, Meibergdreef 9, 1105AZ Amsterdam, The Netherlands; 3 Department of Medical Oncology, Amsterdam UMC, University of Amsterdam, Meibergdreef 9, 1105AZ Amsterdam, The Netherlands; 4 Joint Department of Physics, Institute of Cancer Research and Royal Marsden NHS Foundation Trust, London, UK SM2 5NG UK

**Keywords:** Radiotherapy, MRI, Respiratory motion, Four-dimensional, Cancer, Image quality

## Abstract

**Background:**

For radiotherapy of abdominal cancer, four-dimensional magnetic resonance imaging (4DMRI) is desirable for tumor definition and the assessment of tumor and organ motion. However, irregular breathing gives rise to image artifacts. We developed a outlier rejection strategy resulting in a 4DMRI with reduced image artifacts in the presence of irregular breathing.

**Methods:**

We obtained 2D T2-weighted single-shot turbo spin echo images, with an interleaved 1D navigator acquisition to obtain the respiratory signal during free breathing imaging in 2 patients and 12 healthy volunteers. Prior to binning, upper and lower inclusion thresholds were chosen such that 95% of the acquired images were included, while minimizing the distance between the thresholds (inclusion range (IR)). We compared our strategy (Min95) with three commonly applied strategies: phase binning with all images included (Phase), amplitude binning with all images included (MaxIE), and amplitude binning with the thresholds set as the mean end-inhale and mean end-exhale diaphragm positions (MeanIE). We compared 4DMRI quality based on:

- Data included (DI); percentage of images remaining after outlier rejection.
- Reconstruction completeness (RC); percentage of bin-slice combinations containing at least one image after binning.
- Intra-bin variation (IBV); interquartile range of the diaphragm position within the bin-slice combination, averaged over three central slices and ten respiratory bins.
- IR.
- Image smoothness (S); quantified by fitting a parabola to the diaphragm profile in a sagittal plane of the reconstructed 4DMRI.

A two-sided Wilcoxon’s signed-rank test was used to test for significance in differences between the Min95 strategy and the Phase, MaxIE, and MeanIE strategies.

**Results:**

Based on the fourteen subjects, the Min95 binning strategy outperformed the other strategies with a mean RC of 95.5%, mean IBV of 1.6 mm, mean IR of 15.1 mm and a mean S of 0.90. The Phase strategy showed a poor mean IBV of 6.2 mm and the MaxIE strategy showed a poor mean RC of 85.6%, resulting in image artifacts (mean S of 0.76). The MeanIE strategy demonstrated a mean DI of 85.6%.

**Conclusions:**

Our Min95 reconstruction strategy resulted in a 4DMRI with less artifacts and more precise diaphragm position reconstruction compared to the other strategies.

**Trial registration:**

Volunteers: protocol W15_373#16.007; patients: protocol NL47713.018.14

**Electronic supplementary material:**

The online version of this article (10.1186/s13014-019-1279-z) contains supplementary material, which is available to authorized users.

## Background

Radiotherapy for upper abdominal cancer patients, such as pancreatic, esophageal and gastric cancer, is challenging because of poor contrast between these tumors and other soft tissues on planning CT scans and because of respiratory-induced tumor and organ motion. It was shown that there is a large variation between observers when delineating upper abdominal tumors on computed tomography (CT) [1]. This variation appears to decrease when adding MRI images, which offer better contrast between tumor and the surrounding tissues [1–3]. Respiratory-induced tumor motion, during irradiation is another major challenge for abdominal cancer. Using an internal target volume (ITV), derived from a four-dimensional CT (4DCT), is one way to take this into account. However, for pancreatic cancer it was shown that the initial 4DCT is not representative for the respiratory-induced motion during actual treatment, whilst for esophageal cancer this is the case [4, 5]. Furthermore, to limit X-ray dose burden, the 4DCT acquisition is typically done with a limited number of acquisitions, compromising image quality even further.

4DMRI modalities are promising to overcome the challenges mentioned above, as 4DMRI has superior soft tissue contrast, is flexible, allowing for various image acquisitions, and omits additional imaging radiation dose. A T2-weighted contrast is desirable for tumor and organ at risk visibility [6, 7]. Finally, contrary to CT, MRI offers means for measuring the respiratory signal using internal surrogates, which is shown to be more accurate for amplitude binning strategies compared to the use of external surrogates [8].

One of the challenges of 4DMRI (and 4DCT) is that irregular breathing is known to deteriorate image quality and introduce image artifacts [9–15]. Various strategies of handling irregular breathing have been demonstrated for 4DMRI: e.g., discarding of images associated with an amplitude that falls outside a defined range of amplitudes or applying triggering in order to only obtain images within a certain respiratory amplitude range [11, 16, 17]. However, so far no studies were performed comparing the 4DMRI quality before and after outlier handling. In the presence of irregular breathing, it is vital to balance the exclusion of outliers: including too many data allows outliers to degrade the image quality, whilst excluding too many data potentially leads to the 4DMRI only being representative for a limited percentage of the breathing cycle. Therefore, an important aspect is how the motion of organs and tumors in the reconstructed 4DMRI after outlier handling should be interpreted in radiotherapy.

Ultimately, for radiotherapy treatment planning, a 4DMRI is preferably artifact-free, precise (i.e., the image should accurately depict the anatomy) and give an accurate measure of the respiratory-induced motion during imaging. 4DMRI reconstruction quality should be evaluated on exactly these features. However, most reports on 4DMRI reconstruction quality scored the 4DMRI visually [11]. Some quantitative work has been done, such as fitting a smooth curve to the organ border irregularities and assessing the amount of missing data after retrospective image sorting [13, 18]. Other studies validated 4DMRI reconstruction accuracy with phantom measurements where the reconstructed motions were compared to the physical motion of the phantom [14, 16, 19]. For radiotherapy purposes, all of these quantitative quality measures (i.e., artifacts, precision and validation of reconstruction accuracy) should be addressed.

In this study, we present a 4DMRI acquisition and reconstruction optimized for radiotherapy treatment planning in the abdominal region. We developed a novel outlier rejection strategy and combined it with amplitude binning for robust 4DMRI reconstruction in the presence of irregular breathing. We validated the 4DMRI reconstruction with a dynamic phantom study. Furthermore, by defining a set of 4DMRI quality parameters the precision of the diaphragm position, accuracy of motion amplitude, and the occurrences of image artifacts were quantitatively assessed making it possible to compare 4DMRIs in an objective way. Finally, we compared our outlier rejection strategy and amplitude binning with three other outlier rejection and binning strategies.

## Methods

### 4DMRI reconstruction

**Fig. 1:**
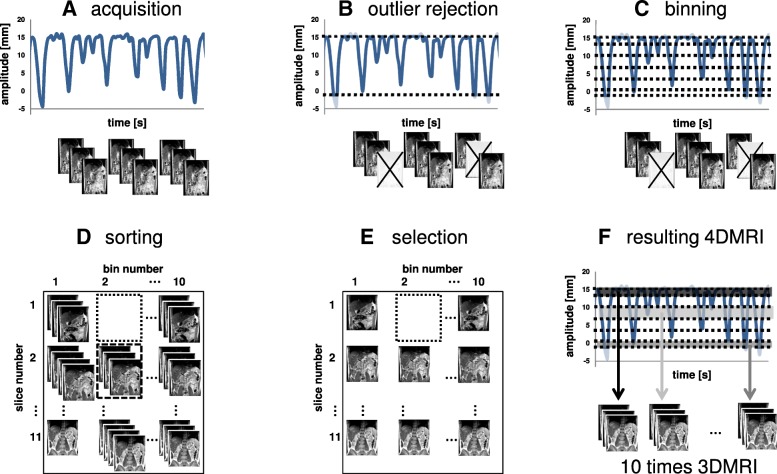
The 4DMRI reconstruction workflow. **a** The respiratory-induced motion of the diaphragm is recorded by use of a 1D navigator during the acquisition of 2D coronal slices as the same volume is repeatedly scanned during free breathing. **b** Outlier rejection is applied. **c** The respiratory signal is binned. **d** The acquired 2D images are associated to a respiratory bin. Empty slice/bin positions may occur (dotted square), affecting reconstruction completeness. Multiple image assignments to one bin-slice position occurs (e.g., dashed square), the image redundancy is exploited to quantify the intra-bin variation. **e** From the bin-slice combinations containing multiple images, the image with the median diaphragm position is selected for 4DMRI reconstruction, resulting in a 4DMRI consisting of 10 respiratory correlated MRI volumes with 11 slices each (**f**)

In the period of March to June 2016, two patient volunteers with cancer in the upper abdomen and twelve healthy volunteers (eight female, four male, mean age 28 years) participated in the study after giving written informed consent in accordance with the medical ethical regulations at our hospital. One patient (P1, male, age 85 years) was treated for metastatic pancreatic cancer and one patient (P2, male, age 61 years) was treated for gastro-esophageal junction cancer. For each subject, a 4DMRI was acquired and reconstructed as described below. Data processing (navigator extraction, binning, sorting, see Fig. 1) was performed off-line using an in-house developed algorithm implemented in MATLAB (MATLAB R2016a, The Math Works Inc., Natick, MA).

#### Acquisition

Image acquisition was performed on a 3.0 T MRI scanner (Ingenia 3.0 T, Philips Healthcare, Best, The Netherlands) using a T2-weighted single-shot turbo spin echo sequence with a field of view of 400 × 200 (superior-inferior × right-left) mm^2^, repetition time of 6061 ms, echo time of 50 ms, and a flip angle of 90 degrees [20]. The bandwidth was 555.9 Hz and sensitivity encoding (SENSE) factor was 4. The sequence and its parameters had been optimized for upper abdominal imaging. Each volume consisted of 11 coronal 2D slices and was acquired repetitively 60 times, i.e., 60 dynamics, during free breathing. The acquired 2D slices had a resolution of 0.78 × 0.78 mm^2^ in-plane and 5 mm slice thickness and were acquired in an interleaved fashion. Image acquisition was interleaved with a 1D navigator, located on the top of the right hemidiaphragm, yielding the diaphragm position every 551 ms. This navigator was used as a respiratory signal surrogate, associating each acquired 2D image with a respiratory state. To correct for geometrical distortions, the 2D gradient non-linearity corrections as provided by the vendor were used. The total scan time was 6 min, obtaining 660 images per data set.

#### Outlier rejection

Irregular breathing deteriorates image quality of the reconstructed 4DMRI which may be mitigated by proper outlier rejection. Furthermore, the representation of respiratory-induced organ motion by the 4DMRI may be defined by outlying respiratory states such as hiccups or a single extra deep inspiration which is in fact not representative for the whole respiratory signal during image acquisition.

We developed an outlier rejection strategy (Min95), applying upper and lower inclusion thresholds over the full respiratory signal, that were chosen such that 95% of the acquired images were included (i.e., 627 images). There are many ways to discard 5% of the data; we chose the thresholds such that the distance between the thresholds (inclusion range, IR) was minimized. As inhale showed more irregular peaks than exhale, this method excluded more outliers at the inhalation side of the respiratory signal. The heuristic choice of 95% resulted in a proper reduction of image artifacts without losing too much data; see Additional file 1: Figure S1 for details.

The percentage of images left for reconstruction after outlier rejection relates to the amount of time that the organ motion is represented by the reconstructed 4DMRI. In other words, if an ITV would be constructed from the 4DMRI with an outlier rejection percentage of 20%, the tumor would be inside the ITV for 80% of the time if the respiration was equal during imaging and treatment. We deemed that 95% was an acceptable percentage for the 4DMRI to be representative for the respiratory-induced organ motion during image acquisition.

#### Binning

**Fig. 2:**
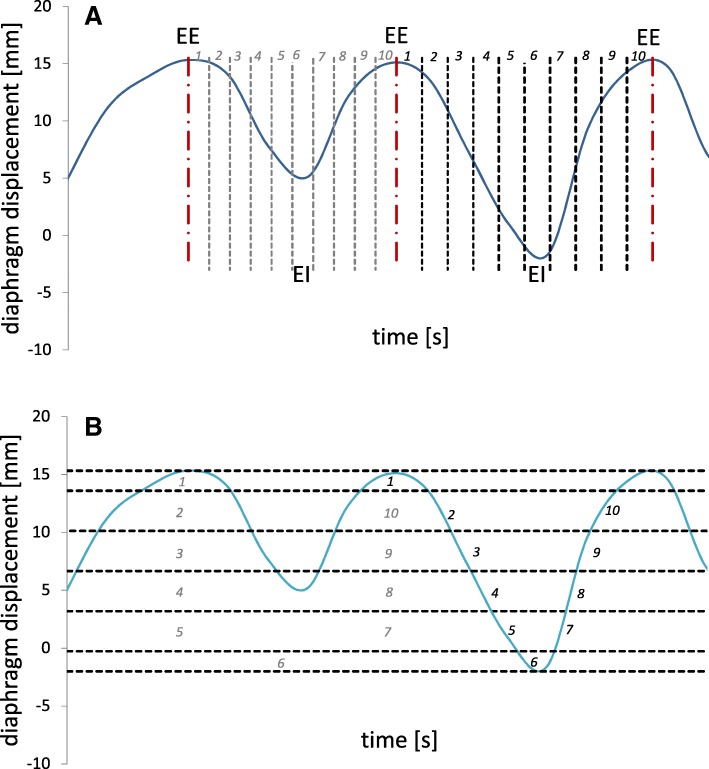
Principle of **a** equidistant phase binning and **b** amplitude binning illustrated for a respiratory signal with breathing irregularities. End-exhale (EE) and end-inhale (EI) positions are indicated

In general, two main binning strategies are used: phase binning and amplitude binning [9, 13–15]. For phase binning, peaks in the respiratory signal are detected and the respiratory cycle is divided in equidistant time bins (Fig. 2a). For amplitude binning, the respiratory signal is binned according to the position of the respiratory surrogate.

In this study we developed an amplitude binning strategy. In our amplitude binning strategy the IR is divided into 5 inhale and 5 exhale amplitude bins (Fig. 2b), covering 6 amplitude ranges. These are defined such that the end-inhale bin (EI) and end-exhale bin (EE) are half the height of the other (equidistant) bins [11].

#### Sorting

The 627 images were retrospectively sorted according to slice position and respiratory state. In our case, with eleven slices per volume and ten respiratory bins, 110 bin-slice combinations needed to be filled. Figure 1d shows a possible outcome of the sorting process. The sampling of the 2D images during acquisition and the periodicity of the respiratory state were uncorrelated. This lead to a random population of bin-slice combinations, i.e. a bin-slice combination can have a single, multiple or no images associated with it.

The choice for acquiring 660 images for 4DMRI reconstruction, or in other terms, to acquire 60 dynamics of the full volume of 11 slices, was made to reduce image artifacts due to missing slices in certain bin-slice combinations. Acquiring more dynamics lowers the chance of missing slices; however, it increases scan time. We optimized the number dynamics and found that the trade-off between missing slices and scan time was optimal at 60 dynamics. Missing bin-slice combinations are not filled, and to visualize a full 3D image an adjacent slice is copied to fill the gap.

The redundancy of multiple images occupying the same bin-slice combination in turn was exploited to quantify image quality (see below). In contrast to the outlier rejection percentage, the interpretation of using one sixth of the available data is time-efficiency of the 4DMRI acquisition.

#### Selection

In the case that multiple images are assigned to one bin-slice combination, one image was selected for 4DMRI reconstruction. For multiple images, variations in diaphragm position between images can be present. The relative location of the diaphragm was quantified by rigid registration of each image to a reference image. The reference image was the first image that was associated to the bin-slice combination. Registration, in cranial-caudal direction only, was based on a manually selected region of interest containing the top of the right hemidiaphragm. We preferred rigid registration over deformable registration since it gives a concise and practically interpretable measure of the 4DMRI reconstruction precision.

The image with the median diaphragm position was selected for 4DMRI reconstruction as we deemed it most representative for the respiratory state. The selection process may introduce an underestimation of the motion amplitude of the diaphragm on the reconstructed 4DMRI.

After the sorting and selection step, up to 110 images from the originally 660 images are used to create 10 volumes of 11 slices each, forming the 4DMRI.

### Phantom study

To validate accuracy and reproducibility of the 4DMRI reconstruction, we performed a phantom study. A moving phantom set-up (Additional file 1: Figure S2) was constructed, attaching a cart (LEGO, Billund, Denmark) via an MR-compatible extension to a Dynamic Thorax Phantom (Computerized Imaging Reference Systems, Incorporated (CIRS), Norfolk, USA). On this cart, a 9.5 cm high tube (diameter 25 mm.) filled with 1% CuSO_4_ aqueous solution was mounted. Prior to acquisition, the physical phantom motion was measured by attaching a pencil to the phantom, letting it draw its trajectory on a sheet of paper.

To simulate a stable breathing pattern, the tube moved periodically over time along the longitudinal axis of the scanner with a position (z) as function of time (t) of z(t) = (A/2)*sin(2π t/T) or z(t) = A*cos^6^(2π t/T) waveform, with A the peak-to-peak amplitude and T the cycle time. The sine waveform was chosen since it is the simplest oscillating function; the cos^6^ function has the form of an ideal respiration without irregularities or outliers. Various input motion signals were used, with A equal to 10, 20, 30 or 40 mm and T equal to 3, 4, or 5 s, see Table 1. For the cos^6^ signal with T = 4 s, the maximum A was reduced to 26 mm by the CIRS motion generation software, as this combination exceeded the maximum speed. To determine reproducibility and precision, acquisition with input signal (sine wave, A = 20 mm, T = 4 s) was repeated four times. MRI data was acquired, and 4DMRI reconstruction with amplitude binning was performed without outlier rejection, since the input motion was unnaturally regular. In this way, the accuracy of the motion on the reconstructed 4DMRI was not influenced by outlier rejection.

**Table 1:** 4DMRI reconstruction validation using phantom measurements. For two waveforms and various cycle times (T), the input amplitude (A) was compared to the motion amplitude from the reconstructed 4DMRIs. The underestimation was the difference between input and measured amplitude. The expected underestimation calculated for the two waveforms is given in the third column. The final column gives the difference between the reconstructed and expected underestimation

Input motion			Reconstructed motion			Calculated expected motion			Difference
Wave form	T	A	A	measured underestimation		A	calculated underestimation		under-estimation
	[s]	[mm]	[mm]	[mm]	[%]	[mm]	[mm]	[%]	[mm]
sin	5	40	37.5	2.5	6.3	38.0	2.0	5.1	0.5
		30	28.1	1.9	6.2	28.5	1.5		0.3
		20	18.8	1.3	6.3	19.0	1.0		0.2
		10	9.4	0.6	6.1	9.5	0.5		0.1
sin	4	40	37.5	2.5	6.3	38.0	2.0	5.1	0.5
		30	28.1	1.9	6.2	28.5	1.5		0.3
		20	18.0	2.0	10.0	19.0	1.0		1.0
		10	9.4	0.6	6.2	9.5	0.5		0.1
sin	3	30	27.3	2.7	8.9	28.5	1.5	5.1	1.1
		20	18.8	1.3	6.3	19.0	1.0		0.2
		10	9.4	0.6	6.2	9.5	0.5		0.1
cos^6^	5	30	28.9	1.1	3.6	29.2	0.8	2.8	0.3
		20	18.8	1.3	6.3	19.4	0.6		0.7
		10	9.4	0.6	6.2	9.7	0.3		0.3
cos^6^	4	26	24.2	1.8	6.8	25.3	0.7	2.8	1.1
		20	19.5	0.5	2.4	19.4	0.6		−0.1
		10	9.4	0.6	6.1	9.7	0.3		0.3
cos^6^	3	20	19.5	0.5	2.3	19.4	0.6	2.8	−0.1
		10	9.4	0.6	6.2	9.7	0.3		0.3
sin	4	20	18.8	1.3	6.3	19.0	1.0	5.1	0.2
		20	18.7	1.3	6.3	19.0	1.0		0.2
		20	18.8	1.3	6.3	19.0	1.0		0.2
		20	18.8	1.2	6.2	19.0	1.0		0.2

To determine the phantom motion for each reconstructed 4DMRI, we used the first bin (i.e., EE) as the reference volume and rigidly registered, in cranial-caudal direction only, each of the remaining nine volumes (VelocityAI, Varian Medical Systems, Atlanta, GA, USA). The maximum displacement observed for the nine volumes was taken as the measured amplitude of the reconstructed 4DMRI. The selection step during 4DMRI reconstruction introduces an underestimation of the measured amplitude of the reconstructed 4DMRI. The expected underestimation was calculated for the sine and cos^6^ waveforms, see Additional file 1 for details.

The measured amplitude of the reconstructed 4DMRI was compared with the input amplitude. The measured underestimation is the difference between these amplitudes. Furthermore, we compared the measured underestimation with the expected underestimation.

### Binning strategy performance

**Fig. 3:**
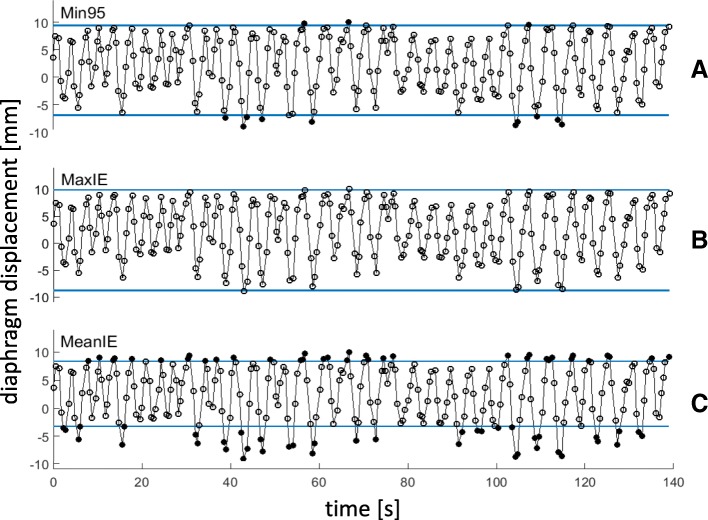
Three amplitude binning strategies, **a** Min95, **b** MaxIE and **c** MeanIE, demonstrated on a section of a typical irregular breathing pattern. The horizontal lines indicate the inclusion thresholds applied for outlier rejection. Open circles: diaphragm positions for which the image was included in the sorting step; filled circles: diaphragm positions for which the images were discarded

**Fig. 4:**
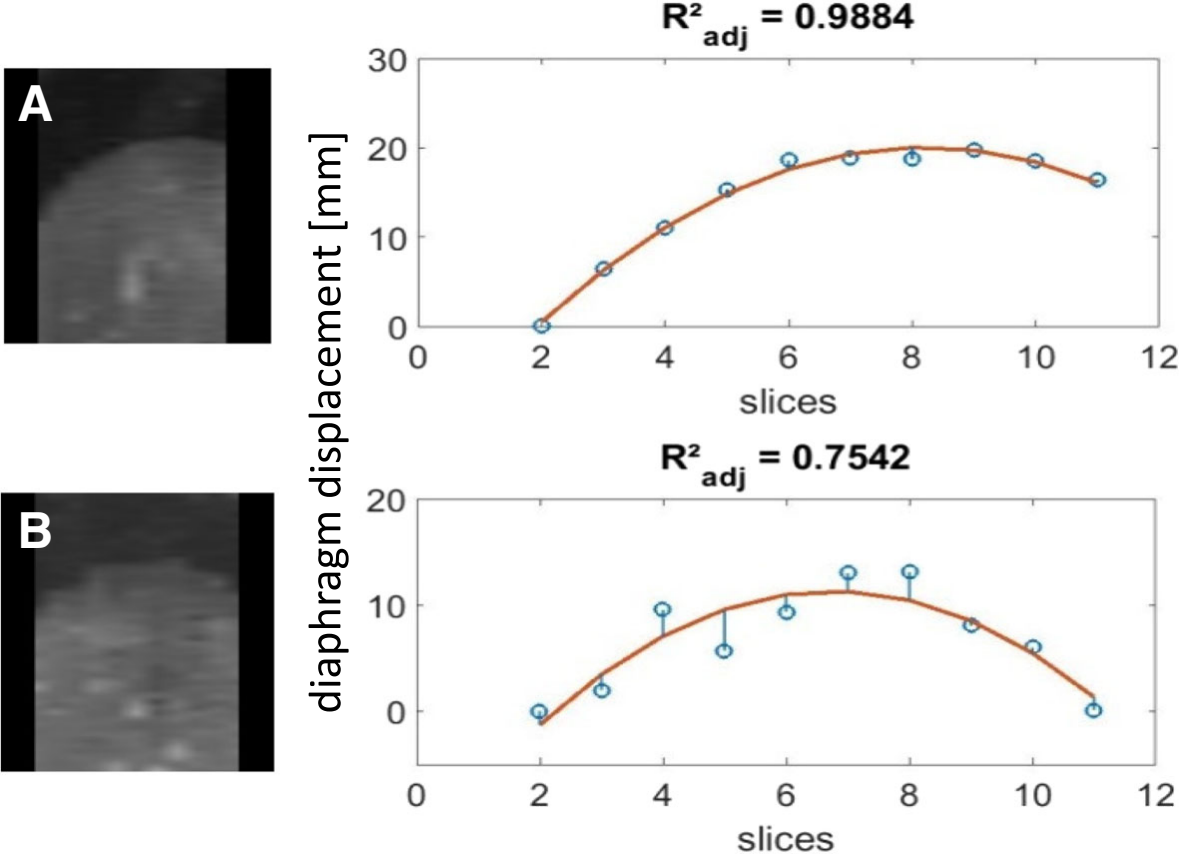
Image smoothness (S) determination. Left shows the sagittal reconstruction of the 3D volume of bin6 of volunteer 11. Right shows the relative position of the diaphragm border with respect to the first slice (open circles) together with the corresponding parabola fit. Residual errors are shown as vertical lines. **a** shows a good S (= R^2^_adj_ averaged over all respiratory bins) of 0.91 reconstructed with the Min95 binning strategy, **b** shows the Phase binning strategy with a poor S of 0.64

The quality of a 4DMRI is defined by its precision, artifact appearance and how reliably it represents the subject’s respiratory-induced motion. In order to quantitatively compare the quality of 4DMRIs, we defined five parameters describing these requirements:

- Data included (DI):
  The percentage of remaining images after outlier rejection (see Fig. 3). This parameter represents the percentage of time in which the motion during image acquisition is represented by the reconstructed 4DMRI.
- Reconstruction completeness (RC):
  The percentage of bin-slice combinations in the 4D data set that contain at least one image (Fig. 1d). For RC < 100%, slices are missing in the reconstructed 4DMRI, giving rise to image artifacts.
- Intra-bin variation (IBV):
  The variation of diaphragm position within a bin-slice combination containing multiple images was quantified by rigid registration (in cranial-caudal direction) of the right hemidiaphragm of the multiple images with respect to one reference image, similar as described in the selection step of 4DMRI reconstruction. The interquartile range (IQR) per respiratory bin of the variation of the diaphragm positions was determined using the diaphragm positions of three (central) slices. Since the bin-slice combinations were randomly populated, a single bin-slice combination may contain too few images to calculate the IQR, for which 4 images are needed at minimum Finally, the IBV was defined as the IQR averaged over all ten respiratory bins. Bin-slice combinations with fewer than four images were not included in the analysis, since the IQR could not be properly calculated for these cases. A high IBV means large diaphragm position variation between images within the bin-slice combinations, indicating a low binning precision.
- Inclusion range (IR):
  The distance between the inclusion thresholds applied for outlier rejection, as depicted in Fig. 3.
- Image smoothness (S):
  Per bin, a parabola was fit to the right diaphragm profile in a sagittal plane of the reconstructed 4DMRI (Fig. 4, left) at the level of the region of interest as chosen for the calculation of the IBV [13]. The diaphragm shape was reconstructed by performing a rigid registration in the cranial-caudal direction of each coronal image with respect to a reference image (i.e. the most ventral slice depicting the diaphragm), see Fig. 4, right. Image smoothness S was defined as the R^2^_adj_ of these fits averaged over all bins. Thus, S ranged from 0 (discontinuous diaphragm; i.e. image artifacts) to 1 (smooth shape; no image artifacts).

For radiotherapy purposes, i.e. target delineation and treatment planning, the optimal 4DMRI has high DI, RC and S and low IBV and IR. For amplitude binning, the IBV and IR should be correlated since the maximal variation of the diaphragm position within one respiratory bin is the bin size. However, for phase binning this is not the case and the IBV can be larger than the bin size. For a respiratory signal with large variations in amplitude, a small IR will decrease DI, yield a smaller IBV and likely a larger S. However, decreasing DI too much will make the 4DMRI less representative.

### Comparison of outlier rejection and binning strategies

4DMRIs were reconstructed for the fourteen subjects. The aforementioned outlier rejection strategy of 95% data inclusion whilst minimizing the inclusion range was combined with amplitude binning, which we defined as the *Min95* strategy, see Fig. 3a. We compared our Min95 4DMRI strategy with three other outlier rejection and binning strategies:

- *Phase*: phase binning without outlier rejection (Fig. 2a). This strategy is commonly used for 4DCT reconstruction [9, 12, 15].
- *MaxIE*: amplitude binning without outlier rejection (Fig. 3b), the lower and upper inclusion thresholds are the outermost EI and EE diaphragm positions.
- *MeanIE*: amplitude binning where the upper inclusion threshold is defined as the mean EE diaphragm position and the lower inclusion threshold as the mean EI diaphragm position (Fig. 3c) [21].

For each strategy, the quality parameters were determined. For each quality parameter the average values and standard deviation over the fourteen subjects were calculated. A two-sided Wilcoxon’s signed-rank test was used to test for significance in differences between the Min95 strategy and the Phase, MaxIE, and MeanIE strategies for the separate parameters. A significance level α = 0.05 was applied.

Note that the 5% outlier rejection strategy can be combined with phase binning as well. However, for this article we chose to limit the number of strategies to the most commonly applied ones.

## Results

### Phantom study

Twenty-three 4DMRIs corresponding to different phantom motions were reconstructed with the MaxIE binning strategy, see Table 1. The amplitude measured with the pencil attached to the phantom matched the input amplitude that was given by the software to the generator (CIRS phantom) with an uncertainty of less than 0.5 mm. Repeatability of the set up was in the submillimeter range of 0.3 mm.

The motion amplitude from the reconstructed 4DMRI showed an underestimation (Table 1). For both waveforms and varying A and T this underestimation was between 0.5–2.7 mm, corresponding to 2.3–10.0% of the input amplitude with an average of 6.1%. For the sin waveform the underestimation was on average 6.7% (6.1–10%) of the input amplitude, for the cos^6^ waveform on average 5.0% (2.3–6.8%).

The expected reconstructed motion underestimation for a sin waveform is 5.1% and of a cos^6^ waveform 2.8%. The measured underestimation of reconstructed motion was within the expected error range taking into account set-up variations, repeatability, and the 0.78 mm in-plane resolution of the MRI images.

### Comparison of outlier rejection and binning strategies

Fourteen subjects were successfully scanned with our MR sequence and 4DMRIs were reconstructed. For all subjects, the breathing signal varied in amplitude during acquisition, see Additional file 1: Figure S4 for details on the respiratory motion and the variation in breathing irregularities between subjects.

**Fig. 5:**
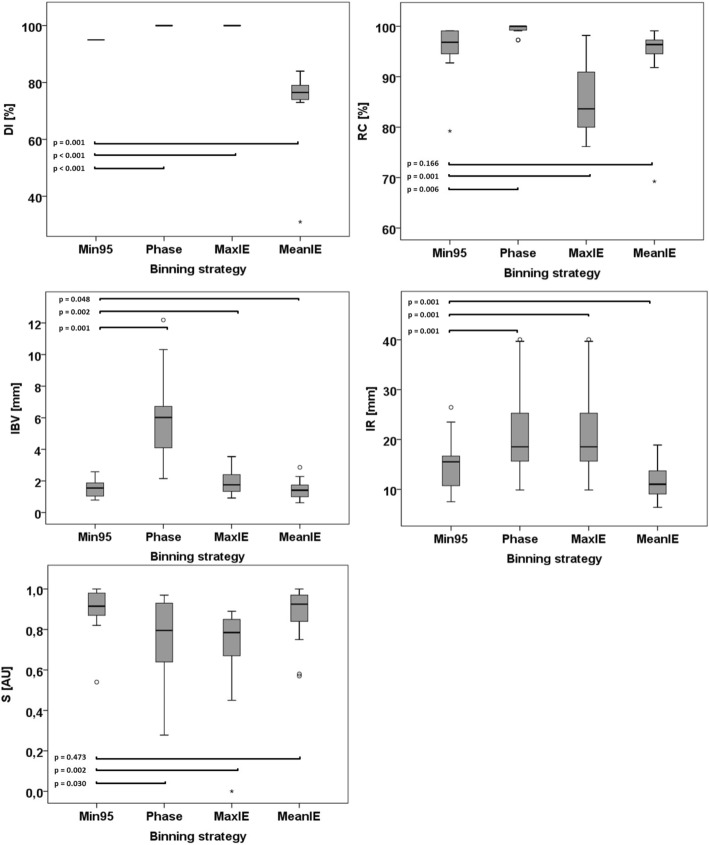
Boxplots of 4DMRI quality parameters for 14 subjects, reconstructed with four binning strategies. Data inclusion (DI), reconstruction completeness (RC), intra-bin variation (IBV), inclusion range (IR) and image smoothness (S). Boxes: median value and lower and higher quartiles; whiskers: lowest and highest data point within 1.5 times the inter-quartile range (IQR) from the quartiles; dots: outliers (outside 1.5 times the IQR from the quartiles), stars: extreme outliers (outside 3 times the IQR from the quartiles). The Min95 technique performed significantly different than each of the other three techniques, except versus MeanIE for RC and S, with α = 0.05

The Min95 strategy was compared to three other strategies (Phase, MaxIE and MeanIE) based on the five quality parameters. Figure 5 shows the distributions of the quality parameters for each strategy. The values of the quality parameters per subject are reported in Additional file 1: Table S1. For volunteer 2, S could not be determined for the MaxIE strategy as fitting failed because of missing images (low RC) in consecutive slices.

For each of the quality parameters, comparing the Min95 strategy with the Phase, MaxIE, and MeanIE strategies showed significant differences for all but RC (Min95 vs. MeanIE *p* = 0.166) and S (Min95 vs. MeanIE *p* = 0.473). The Min95 strategy outperformed the other three binning strategies. It showed least image artifacts with a mean S of 0.90 and a high mean reconstruction completion of 95.5%, while it had low mean IBV of 1.6 mm.

Phase binning and MaxIE showed the largest mean IR (21.3 mm) due to the absence of outlier rejection and had poorest mean S of 0.76. For Phase binning the low S was because of the large mean IBV of 6.3 mm, with a high RC of 99.5%. For the MaxIE strategy the image artifacts were caused by the poor mean RC of 85.6% due to empty bin-slice combinations for the EI respiratory bins. However, the IBV was relatively low (1.9 mm). The MeanIE strategy did not suffer from high IBV and low RC, and therefore showed few image artifacts (mean S of 0.87) with low mean IBV of 1.5 mm. However, the rigorous outlier rejection resulted in a mean DI of only 74%, meaning that the resulting 4DMRI only described the motion of the anatomy for 74% of the time. The Min95 strategy had virtually the same IBV, RC, and S as the MeanIE strategy, with the advantage of having a high DI of 95%.

Outlier rejection resulted in smaller standard deviations for most of the quality parameters, e.g. the standard deviation of IR of the Min95 strategy was 5.7 mm compared to 9.8 mm for Phase and MaxIE, the standard deviation in IBV was 0.6 mm for Min95 compared to 2.7 mm for Phase. This indicated that proper handling of respiratory outliers resulted in a more robust 4DMRI reconstruction in the presence of irregular breathing.

**Fig. 6:**
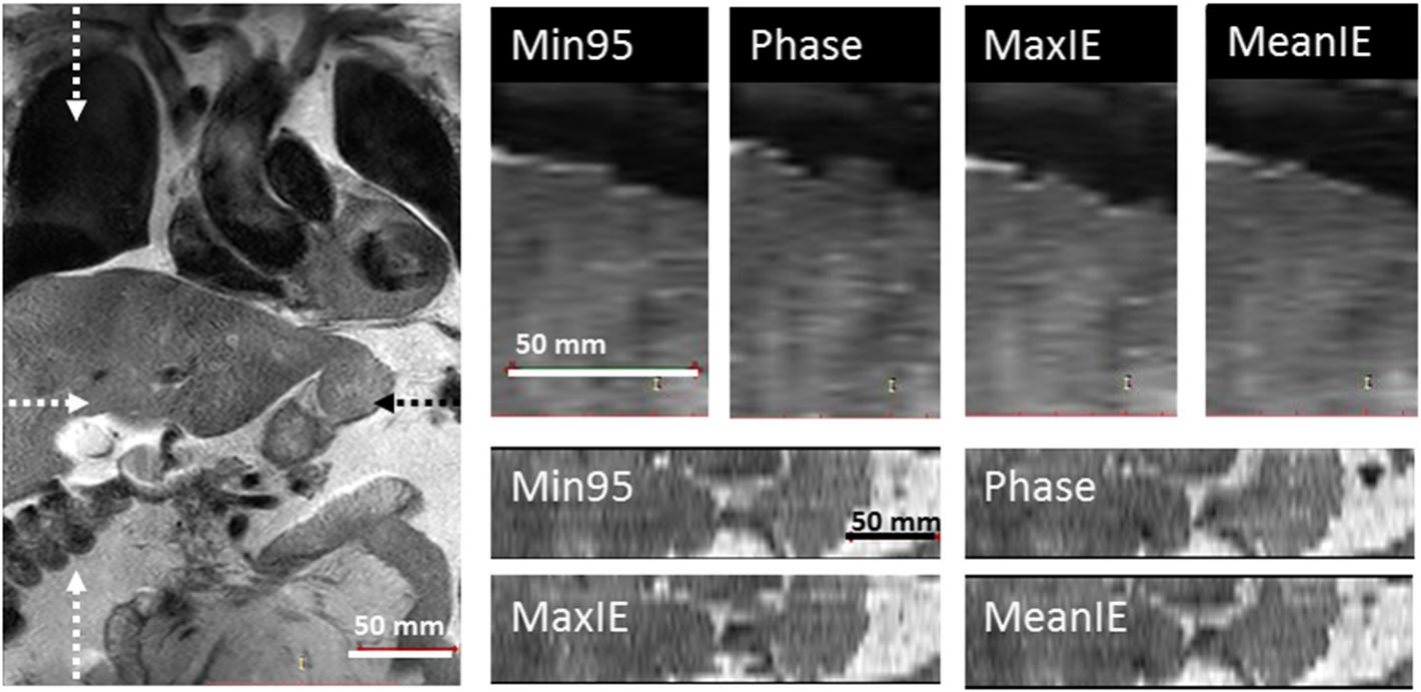
Example 4DMRIs (bin 5) reconstructed for Patient 2 treated for gastro-esophageal cancer. Left: The acquired coronal image of slice position 6; upper right: sagittal view of the diaphragm reconstructed with four binning strategies (Min95, Phase, MaxIE, and MeanIE); lower right: axial views on the level of the pancreas. Dotted arrows in the coronal plan depict the location of the sagittal and axial planes. Artifacts, e.g. poor diaphragm smoothness with a jittery appearance appear for the Phase and the MaxIE outlier rejection strategy in both axial and sagittal planes

Figure 6 illustrates the findings of the quality parameters for a representative clinical case of a gastroesophageal junction cancer patient. In the sagittal plane, the Phase strategy showed a rather poor diaphragm smoothness with artifacts appearing as discontinuities in the sagittal reconstruction because of the assignment of images with large anatomical variations (i.e., large IBV) to the same respiratory bin. The same could be seen for the MaxIE strategy where the diaphragm discontinuities resulted from a low RC. The DICOM viewer (Radiant version 4.2.1, Poznán, Poland) used in the picture fills the missing slice with a copy of a neighboring slice. The MeanIE and Min95 strategies showed a smooth diaphragm profile. The axial reconstructions showed similar features; the diaphragm dome showed jittery borders for Phase binning and MaxIE, though MeanIE and Min95 had smooth borders. In a subtle fashion, the difference in IR was visible between the sagittal reconstruction of the MeanIE and Min95 strategies, where the MeanIE showed a lower diaphragm position for the EI respiratory phase.

**Fig. 7:**
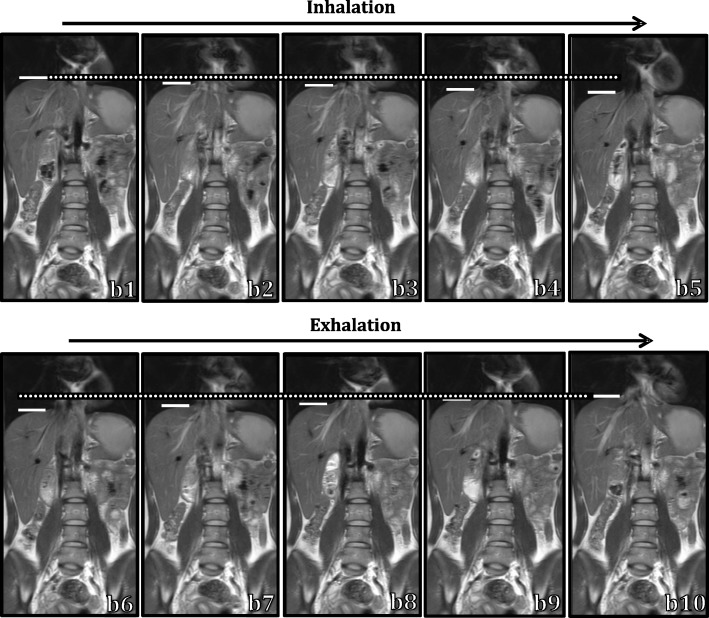
Example 4DMRI of volunteer 2 (chosen for its large motion amplitude for visibility), reconstructed with the Min95 strategy. One breathing cycle is displayed by coronal images of slice 7 from bin b1 - b10. The white bars indicate the top of the right hemidiaphragm, showing the respiratory-induced diaphragm motion

Figure 7 shows the images over the respiratory cycle, depicting one coronal slice of a 4DMRI of a volunteer with a large breathing motion amplitude.

## Discussion

We presented a 4DMRI acquisition and reconstruction approach, with robust and precise 4DMRI reconstruction (i.e., fewer artifacts) in the presence of irregular breathing compared to other binning strategies. The acquisition time of 6 min is suitable for clinical routine and provides information about patient-specific breathing motion over extended duration. Validation and quality assessment of the produced 4DMRIs was provided in terms of diaphragm position precision and artifact occurrences. This is the first 4DMRI study providing quantitative assessment of reconstruction precision, image artifacts, and motion validation comparing different binning and outlier rejection strategies, supplemented with a phantom validation study.

Commercially available MRI sequences were used, followed by an in-house developed post-processing technique that sorted scanner-reconstructed images. Thus, adaptations at the scanner such as customized sequences and patched software installations were not required. As a result, the image quality was up to clinical standard and can be used in clinical routines. The resulting images provided information on patient specific respiratory-induced motion, which can be incorporated in radiotherapy, e.g. by using a personalized internal target volume definition or algorithms for mid-ventilation techniques [22, 23]. The superior soft tissue contrast, optimized for imaging organs in the abdomen, and high quality images (few artifacts, precise imaging) may reduce delineation uncertainties compared to CT scans [1, 2], potentially reducing planning target volumes and thereby healthy tissue irradiation and corresponding toxicities.

Twelve healthy volunteers and two patients with cancer in the upper abdomen were included in this study. Although the volunteer cohort might not entirely represent the patient population’s respiratory characteristics and its variation, the volunteer cohort showed a large variety of breathing irregularities (see Additional file 1: Figure S4).

The presented 4DMRI acquisition consists of coronal images, since this depicts the diaphragm dome optimally, allowing for quality assessment of the resulting 4DMRI. The use of the well-established T2-weighted single shot turbo spin echo imaging benefits from high in-plane resolution. Furthermore, each slice was acquired in a short time span of 551 ms per slice providing a sharp snapshot of the anatomy during free breathing. In this study the diaphragm position in the coronal slices was used for median image selection. This could have been done on diaphragm position extracted from the 1D navigator as well, enabling the 4DMRI reconstruction as described in this manuscript to be done for transversal and sagittal slices as well.

The presented 4DMRI acquisition and reconstruction is a result of the requirements of radiotherapy and has advantages and disadvantages compared to other reported 4DMRI methods. The choice for adopting retrospective binning is analogous to conventional 4DCT, which facilitates implementation in current clinical practice. In contrast to 4DCT, 4DMRIs can be reconstructed in many more ways other than using an external surrogate for registering the respiratory signal. For instance, retrospective binning in k-space provides an elegant means to reconstruct 3D volumes by combining acquisitions of k-lines from different respiratory cycles, benefiting from fast acquisition and high signal-to-noise images [8]. However, these k-space sorted 4DMRI techniques are limited to steady-state sequences, limiting the contrast to T1-weighted or T2/T1-weighted in most cases [24, 25]. To have more T2-like weighted (T2W) contrast requires more complex steady-state sequences not commonly used for abdominal imaging [6]. Alternatively, the deformation field from a k-space sorted T1-weighted 4DMRI can be applied to a 3D T2W contrast image [26]. An improvement in 2D reconstruction could be achieved by acquiring 2D images perpendicular to the coronal planes and apply a super-resolution reconstruction method in order to reconstruct high-resolution 4DMRI with T2W contrast [27]. Furthermore, evaluating the precision of the resulting 4DMRIs cannot be done on for instance diaphragm shape artifacts (i.e. S), relying on a 2D acquisition technique rather than a 3D acquisition. The outlier rejection strategy described in this work may also be applied for k-space binning, potentially improving the image quality further. However, as most k-space binning approaches rely on central k-space magnitude instead of absolute diaphragm position, the technique might need slight adapting for best results.

A 1D navigator has been shown to be a better surrogate for abdominal organ motion than an external surrogate [8]. For amplitude binning, correspondence of surrogate displacement with organ displacement is more vital than for phase binning. For this reason, the use of a navigator instead of external surrogates [14, 16, 28] to determine the respiratory signal was preferred for amplitude binning based 4DMRI reconstruction. Self-navigation, e.g. using the diaphragm position on the acquired 2D images for breathing registration is a promising option though outside of the scope of this study [29, 30].

The presented scanning sequence acquired 60 dynamics (i.e., repetitions of the scanned volume) in 6 min. When sorting the images, a bin-slice combination may contain multiple images; this we exploited in quantifying the reconstruction precision. However, by selecting the image with the median diaphragm position as the image for 4DMRI reconstruction, an underestimation of motion amplitude was introduced. This underestimation was quantified by phantom measurements and was conform the calculated expected underestimation. For 4DMRI, similar errors in measured amplitude are reported, and in a phantom experiment a similar relative motion amplitude underestimation of 5% was found for 4DCT [15, 25].

With the presented 4DMRI reconstruction method in clinical practice, motion amplitude underestimation will occur and can be expected to be in the range of 0.6–5.4% of the motion amplitude if one uses 10 respiratory bins and assumes that the peak and valley shape are between very sharp (i.e., the peak of a cos^6^ waveform) or rather flat (e.g., the plateau of a cos^6^ waveform), see Additional file 1: Figure S3. Using more and smaller respiratory bins for 4DMRI reconstruction, which may come at a price of lower RC or longer scan times, reduces the motion amplitude underestimation.

Our novel amplitude binning strategy adopted outlier rejection, preventing that outlier positions define the range over which amplitude binning was performed, which would result in poor RC or IBV. We chose to discard 5% of the data to mitigate the largest effect of irregular breathing, resulting in a mild underestimation of motion, directly related to the fraction of time the 4DMRI is in fact correctly depicting the organ motion trajectory. On average, discarding 5% of the data resulted in a 28% smaller IR, which may lead to a smaller patient-specific internal target volume, potentially sparing healthy tissue. The ITV is designed to cover the entire tumor trajectory, though this assumes that the 4D imaging provides the correct tumor motion. Irregular breathing such as shown in Additional file 1: Figure S4, deteriorates 4DMRI image quality, and potentially gives rise to unnecessary large treatment volumes when (unrepresentative) outlier tumor positions due to hiccups or a single deep inspiration are included. Therefore, not applying outlier rejection is not the optimal reconstruction strategy for both image quality and target definition. The dosimetric and clinical effect of excluding 5% of the data needs to be investigated and is a study on its own, as we focused on the quality of the reconstructed images. However, not covering the target for a certain part of the time during treatment in order to spare a large volume of healthy tissue is an accepted paradigm in radiotherapy as it is the base of treatment margin recipes [31].

For radiotherapy treatment planning purposes, a 4DMRI should be precise, artifact-free and the reconstructed respiratory motion amplitude should be representative for the motion during the scan. The quality of 4DMRI datasets as measured by the set of quality parameters was significantly higher for the novel Min95 binning strategy compared to phase binning and the two other amplitude binning strategies. 4DMRI reconstruction was precise with a mean IBV of 1.6 mm, had smooth diaphragm profiles (S of 0.90), had a small inclusion range and did not suffer much from missing slices (RC of 95.5). It performed as good as applying strict outlier reduction (MeanIE) without the severe drawback of heavily underestimating the organ motion [21]. Furthermore, this improvement was present for all subjects and the variation between subjects was reduced as well, indicating that the novel amplitude binning technique is robust against variation in patterns of irregular breathing. Each strategy has its strong points and weak points, e.g. a method that does not reject any outliers will result in a 100% DI although consequently it will suffer from artifacts resulting from low RC or high IBV, since in the case of irregular breathing, the chance that during sorting bin-slice combinations will not be filled increases (See Additional file 1: Figure S1). Choosing the optimal strategy will therefore be a well-argued balance between the performance of the various quality parameters.

4DMRI shows the tumor position in various respiratory phases. This can be integrated in a radiotherapy procedure similar to how 4DCT is used for lung and esophageal cancer treatment [32, 33]. In case this 4DMRI technique is used for target volume delineation, the underestimation of the reconstructed motion should be taken into account. How to incorporate this into an ITV, or in a PTV margin in case a mid-position MRI is reconstructed from the 4DMRI, needs to be investigated in a future study. Furthermore, the described field of view is limited and needs to be expanded to encompass larger anatomical structures. This comes at a cost of scanning time. Reducing scanning time by acquiring fewer dynamics will potentially increase the incidence of missing bin-slice combinations and introduce a lower RC. A limitation of our reconstruction strategy is that we do not fill up missing bin-slice combinations when the RC is not 100%. A high RC still results in clinically usable 4DMRI since all anatomical structures and motion are still properly reconstructed. When the RC drops below 90% and the missing slices are in unfortunate positions (e.g., adjacent slices in the end-inhale and end-exhale bins), the quality of the 4DMRI deteriorates. A post-processing step such as interpolation of adjacent slices or adjacent respiratory bins might fill the missing bin-slice combinations, enabling for speeding up the image acquisition by lowering the number of dynamics [30, 34]. However, a high RC will be preferable to such post-processing steps.

Acquiring a dataset pre-treatment for treatment planning purposes does not guarantee that the same (irregular) breathing motion will be present during the multiple radiotherapy treatment sessions. It is known that abdominal organ motion measured on pre-treatment imaging can be different from motion observed during treatment sessions [4]. This is independent of the imaging technique, and as such present for 4DCT as well as in current clinical practice [35]. However, compared to 4DCT the presented 4DMRI is acquired over an extended time of 6 min, increasing the possibility that the reconstructed motion of 4DMRI after outlier rejection is more representative than the motion observed with 4DCT which would take 2 min over such a field of view.

## Conclusion

The novel amplitude binning strategy discarding outliers results in 4DMRIs that are precise and have few artifacts, showing potential towards clinical application in radiotherapy for abdominal cancer. The defined set of quality parameters is suited for explicit validation of 4D image quality and enables a quantitative comparison between different 4DMRI reconstruction techniques. In a comparison based on these quality parameters, our strategy outperformed the three other commonly used 4DMRI binning and outlier rejection strategies with higher precision of the diaphragm position and fewer image artifacts.

## Additional file

Additional file 1: Determination of the optimal percentage of outlier exclusion, pictures of the phantom measurements set-up, the calculation of the expected underestimation for a cos^6^ breathing pattern, the breathing patterns per subject, and the quality parameters values for each subject for each of the described outlier rejection and binning strategies. (PDF 1903 kb)

## Abbreviations

4DCT: Four-dimensional CT
4DMRI: Four-dimensional MRI
CT: Computed tomography
DI: Data included
EE: End-exhale
EI: End-inhale
IBV: Intra-bin variation
IQR: Interquartile range
IR: Inclusion range
ITV: Internal target volume
MRI: Magnetic resonance imaging
S: Image smoothness
T2W: T2-weighted

## References

[1] Gurney-Champion OJ, Versteijne E, van der Horst A (2017). Addition of MRI for CT-based pancreatic tumor delineation: a feasibility study. Acta Oncol.

[2] Versteijne E, Gurney-Champion OJ, van der Horst A (2017). Considerable interobserver variation in delineation of pancreatic cancer on 3DCT and 4DCT: a multi-institutional study. Radiat Oncol.

[3] Villeirs GM, Van Vaerenbergh K, Vakaet L (2005). Interobserver delineation variation using CT versus combined CT + MRI in intensity-modulated radiotherapy for prostate cancer. Strahlenther Onkol.

[4] Lens E, van der Horst A, Kroon PS (2014). Differences in respiratory-induced pancreatic tumor motion between 4D treatment planning CT and daily cone beam CT, measured using intratumoral fiducials. Acta Oncol.

[5] Jin P, Hulshof M, van Wieringen N, Bel A, Alderliesten T (2017). Interfractional variability of respiration-induced esophageal tumor motion quantified using fiducial markers and four-dimensional cone-beam computed tomography. Radiother Oncol.

[6] Gurney-Champion OJ, Nederveen AJ, Klaassen R (2016). Revisiting the potential of alternating repetition time balanced steady-state free precession imaging of the abdomen at 3 T. Investig Radiol.

[7] Du D, Caruthers SD, Glide-Hurst C (2015). High-quality t2-weighted 4-dimensional magnetic resonance imaging for radiation therapy applications. Int J Radiat Oncol Biol Phys.

[8] Stemkens B, Tijssen RHN, de Senneville BD (2015). Optimizing 4-dimensional magnetic resonance imaging data sampling for respiratory motion analysis of pancreatic tumors. Int J Radiat Oncol Biol Phys.

[9] Abdelnour AF, Nehmeh SA, Pan T (2007). Phase and amplitude binning for 4D-CT imaging. Phys Med Biol.

[10] Cai J, Chang Z, Wang Z, Paul Segars W, Yin F-F (2011). Four-dimensional magnetic resonance imaging (4D-MRI) using image-based respiratory surrogate: a feasibility study. Med Phys.

[11] Li G, Wei J, Olek D, Kadbi M, Tyagi N, Zakian K, Mechalakos J, Deasy JO, Hunt M. Direct comparison of respiration-correlated four-dimensional magnetic resonance imaging (4DMRI) reconstructed based on concurrent internal navigator and external bellows. Int J Radiat Oncol Biol Phys. 2016;97:596–605. 10.1016/j.ijrobp.2016.11.004 PMC5288126 28011048

[12] Olsen JR, Lu W, Hubenschmidt JP (2008). Effect of novel amplitude/phase binning algorithm on commercial four-dimensional computed tomography quality. Int J Radiat Oncol Biol Phys.

[13] Paganelli C, Summers P, Bellomi M, Baroni G, Riboldi M (2015). Liver 4DMRI: a retrospective image-based sorting method. Med Phys.

[14] Tokuda J, Morikawa S, Haque HA (2008). Adaptive 4D MR imaging using navigator-based respiratory signal for MRI-guided therapy. Magn Reson Med.

[15] Wink N, Panknin C, Solberg TD (2006). Phase versus amplitude sorting of 4D-CT data. J Appl Clin Med Phys..

[16] Deng Z, Pang J, Yang W (2016). Four-dimensional MRI using three-dimensional radial sampling with respiratory self-gating to characterize temporal phase-resolved respiratory motion in the abdomen. Magn Reson Med.

[17] Yue Y, Fan Z, Yang W (2015). Geometric validation of self-gating k-space-sorted 4D-MRI vs 4D-CT using a respiratory motion phantom. Med Phys.

[18] van de Lindt TN, Sonke JJ, Nowee ME (2018). A self-sorting coronal 4D-MRI method for daily image guidance of liver lesions on an MR-Linac. Int J Radiat Oncol Biol Phys.

[19] Stemkens B, Tijssen RH, de Senneville BD, Lagendijk JJ, van den Berg CA (2016). Image-driven, model-based 3D abdominal motion estimation for MR-guided radiotherapy. Phys Med Biol.

[20] Tekelenburg D, Gurney-Champion O, Lens E (2016). PO-0913: clinically applicable T2-weighted 4D magnetic resonance imaging with good abdominal contrast. Radiother Oncol.

[21] Fitzpatrick MJ, Starkschall G, Antolak JA (2006). Displacement-based binning of time-dependent computed tomography image data sets. Med Phys.

[22] Peulen H, Belderbos J, Rossi M, Sonke JJ (2014). Mid-ventilation based PTV margins in stereotactic body radiotherapy (SBRT): a clinical evaluation. Radiother Oncol.

[23] van Herk M, Witte M, van der Geer J, Schneider C, Lebesque JV (2003). Biologic and physical fractionation effects of random geometric errors. Int J Radiat Oncol Biol Phys.

[24] Rank CM, Heusser T, Buzan MT (2017). 4D respiratory motion-compensated image reconstruction of free-breathing radial MR data with very high undersampling. Magn Reson Med.

[25] Han F, Zhou Z, Cao M, Yang Y, Sheng K, Hu P (2017). Respiratory motion-resolved, self-gated 4D-MRI using rotating cartesian k-space (ROCK). Med Phys.

[26] Freedman JN, Collins DJ, Bainbridge H (2017). T2-weighted 4D magnetic resonance imaging for application in magnetic resonance-guided radiotherapy treatment planning. Investig Radiol.

[27] Freedman JN, Collins DJ, Gurney-Champion OJ, McClelland JR, Nill S, Oelfke U, Leach MO, Wetscherek A. Super-resolution T2-weighted 4D MRI for image guided radiotherapy. Radiother Oncol. 2018;129:486–93. 10.1016/j.radonc.2018.05.015 PMC6294732 29871813

[28] Buerger C, Clough RE, King AP, Schaeffter T, Prieto C (2012). Nonrigid motion modeling of the liver from 3-D undersampled self-gated golden-radial phase encoded MRI. IEEE Trans Med Imaging.

[29] Fontana G, Riboldi M, Gianoli C (2016). MRI quantification of pancreas motion as a function of patient setup for particle therapy -a preliminary study. J Appl Clin Med Phys.

[30] van de Lindt TN, Fast MF, van der Heide UA, Sonke JJ. Retrospective self-sorted 4D-MRI for the liver. Radiother Oncol. 2018;127:474–80. 10.1016/j.radonc.2018.05.006 29804801

[31] van Herk M (2004). Errors and margins in radiotherapy. Semin Radiat Oncol.

[32] Wolthaus JW, Schneider C, Sonke JJ (2006). Mid-ventilation CT scan construction from four-dimensional respiration-correlated CT scans for radiotherapy planning of lung cancer patients. Int J Radiat Oncol Biol Phys.

[33] Chen X, Lu H, Tai A, Johnstone C, Gore E, Li XA (2014). Determination of internal target volume for radiation treatment planning of esophageal cancer by using 4-dimensional computed tomography (4DCT). Int J Radiat Oncol Biol Phys.

[34] Garcia D (2010). Robust smoothing of gridded data in one and higher dimensions with missing values. Comput Stat Data Anal.

[35] Ge J, Santanam L, Noel C, Parikh PJ (2013). Planning 4-dimensional computed tomography (4DCT) cannot adequately represent daily intrafractional motion of abdominal tumors. Int J Radiat Oncol Biol Phys.

