# Supplementary material for: A novel amplitude binning strategy to handle irregular breathing during 4DMRI acquisition: improved imaging for radiotherapy purposes
Source: Radiat Oncol. 2019 May 14;14:80. doi: 10.1186/s13014-019-1279-z (PMC6518684; doi:10.1186/s13014-019-1279-z)
Supplement: Supplementary file 1 — Determination of the optimal percentage of outlier exclusion, pictures of the phantom measurements set-up, the calculation of the expected underestimation for a cos6 breathing pattern, the breathing patterns per subject, and the quality parameters values for each subject for each of the described outlier rejection and binning strategies. (PDF 1903 kb) [file 13014_2019_1279_MOESM1_ESM.pdf]

Additional file 1

A novel amplitude binning strategy to handle irregular breathing during 4DMRI acquisition: improved imaging for radiotherapy purposes

Z. van Kesteren *et al.*

Determination of the percentage of outlier exclusion

Choosing the optimal percentage of included data for the Min95 outlier rejection strategy with amplitude binning was done by investigating the influence of the percentage of data included (DI) on the 4DMRI quality, using the acquired images of the first five volunteers and the two patient cases. We sampled percentages from 80% to 100% in steps of 5%, evaluating the quality of the resulting 4DMRI according to the quality parameters described in the manuscript. For each DI, the minimal inclusion range (IR) was chosen.

Figure S1 shows DI versus reconstruction completeness (RC), IR, and intra-bin variation (IBV). Overall, RC was low when using a DI of 100%, improving greatly when excluding 5% of the data. Reducing the DI further did not improve the RC further. Similar behavior was observed for the IR relation with respect to DI, where exclusion of 5% of the data yielded the largest reduction in IR, showing that IR is dominated by a few outlier positions. For the IBV, no clear cutoff point could be identified. For the amplitude binning strategies the IBV is expected to be correlated to the IR, since its maximum value is the bin size. The IBV can be larger than the bin size for phase binning, because diaphragm positions from different lung volumes can be assigned to the same respiratory bin.

Based on this, we fixed DI at 95% for the amplitude binning strategy, hence called the Min95 strategy.

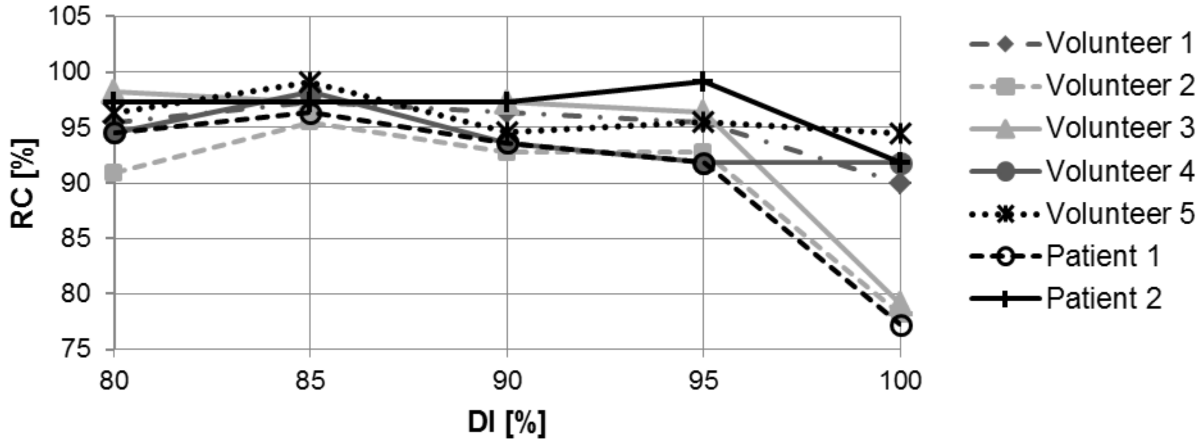

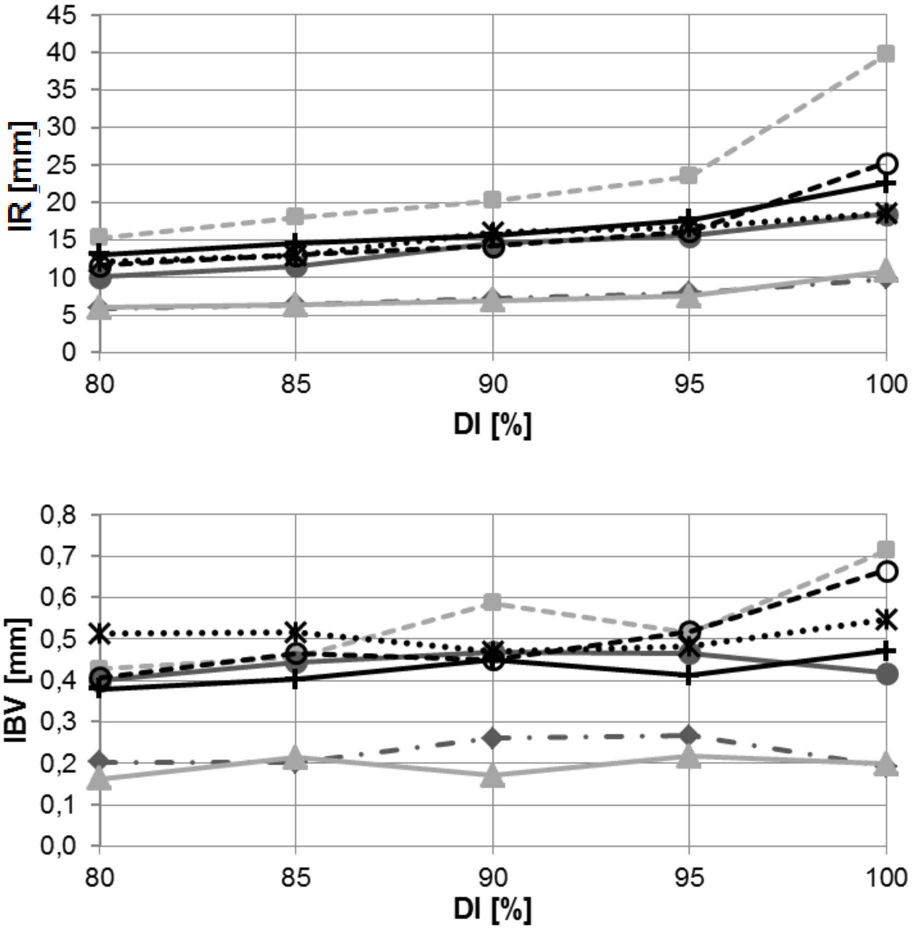

Figure S1: *Influence of the amount of outlier rejection, or data included (DI), on reconstruction completeness (RC), intra-bin variation (IBV), and inclusion range (IR) of 4DMRIs reconstructed with amplitude binning, for 7 subjects.*

Phantom measurement set-up

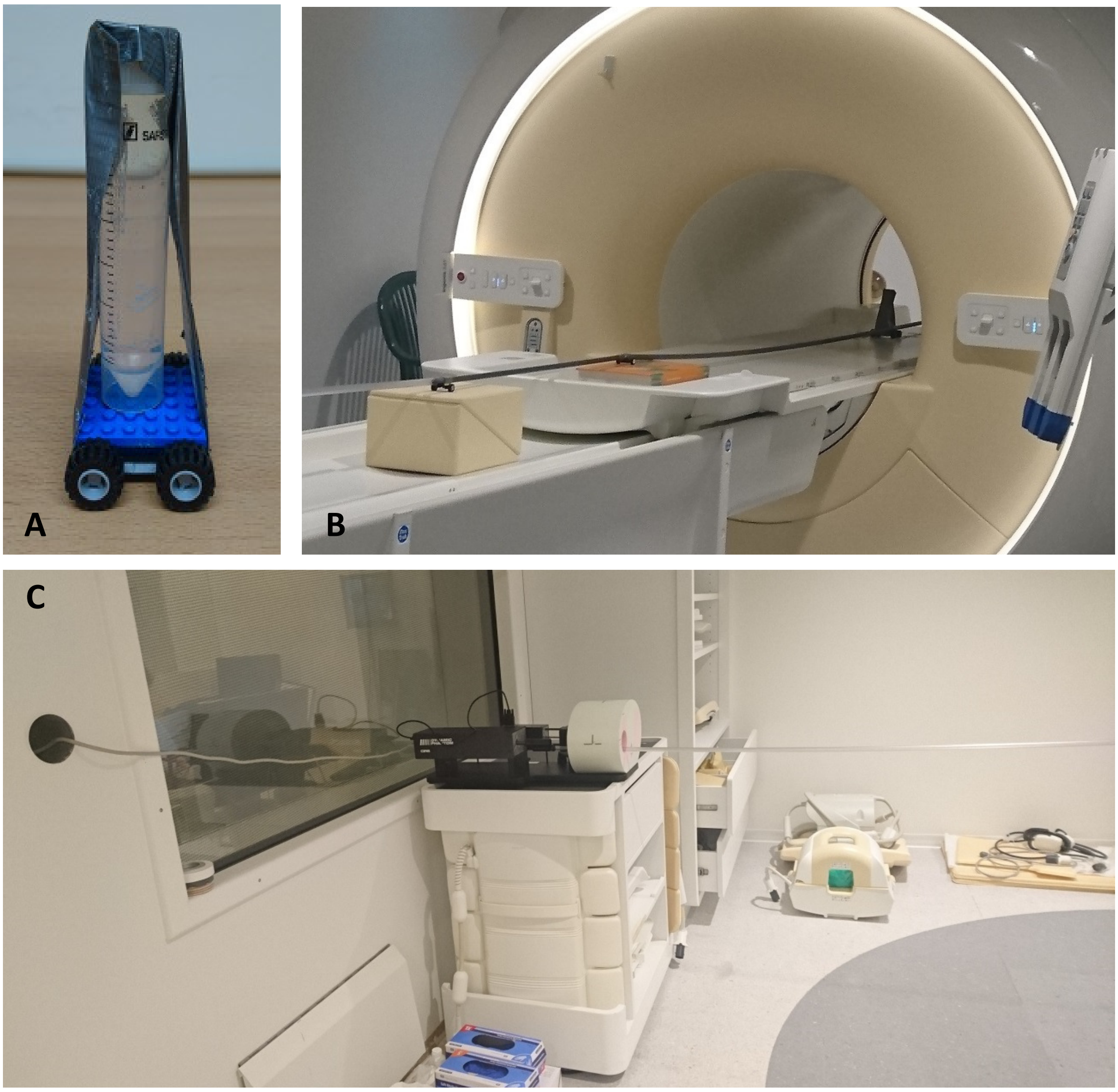

Figure S2: *Phantom study set-up. (A) MR compatible phantom consisting of a tube, filled with 1% $CuSO_4$ aqueous solution, fixed onto a Lego cart. (B) The phantom in the scanner bore, connected to the motion translating extension. Extension rods are supported to avoid slack from generator to phantom. To reduce friction between extension rods and supporting elements, they are guided via Lego wheels. (C) CIRS Dynamic Thorax Phantom; motion generator with extension for motion translation to the phantom.*

Expected underestimation of reconstructed motion amplitude

The presented 4DMRI reconstruction contains a selection procedure for when a bin-slice combination contains multiple 2D images. Each image may have a different diaphragm position. The image with the median diaphragm position is selected for 4DMRI reconstruction; this introduces an underestimation in reconstructed motion amplitude of the diaphragm since the images with the extreme positions are not used for reconstruction.

For a regular periodic waveform, this underestimation can be calculated. For the validation of the 4DMRI reconstruction technique, phantom measurements with a $\cos^6$ waveform were

used. The expected underestimation was calculated as follows: at the end-exhale and end-inhale positions, the bin height is 10% of the amplitude. Each bin spans a certain time, the median time point is defined as the point in the middle of the bin. For the end-inhalation bin this corresponds to t = ½ acos($\sqrt[6]{0.9}$), for the end-exhalation bin to t = $\frac{\pi}{4}$ + ½ acos($\sqrt[6]{0.1}$). Corresponding median amplitudes for a $\cos^6$ waveform are end-inhale (EI) = 97.4% and end-exhale (EE) = 0.2%. The total underestimation adds up to 2.8%. For a sin waveform the total underestimation adds up to 5.1%.

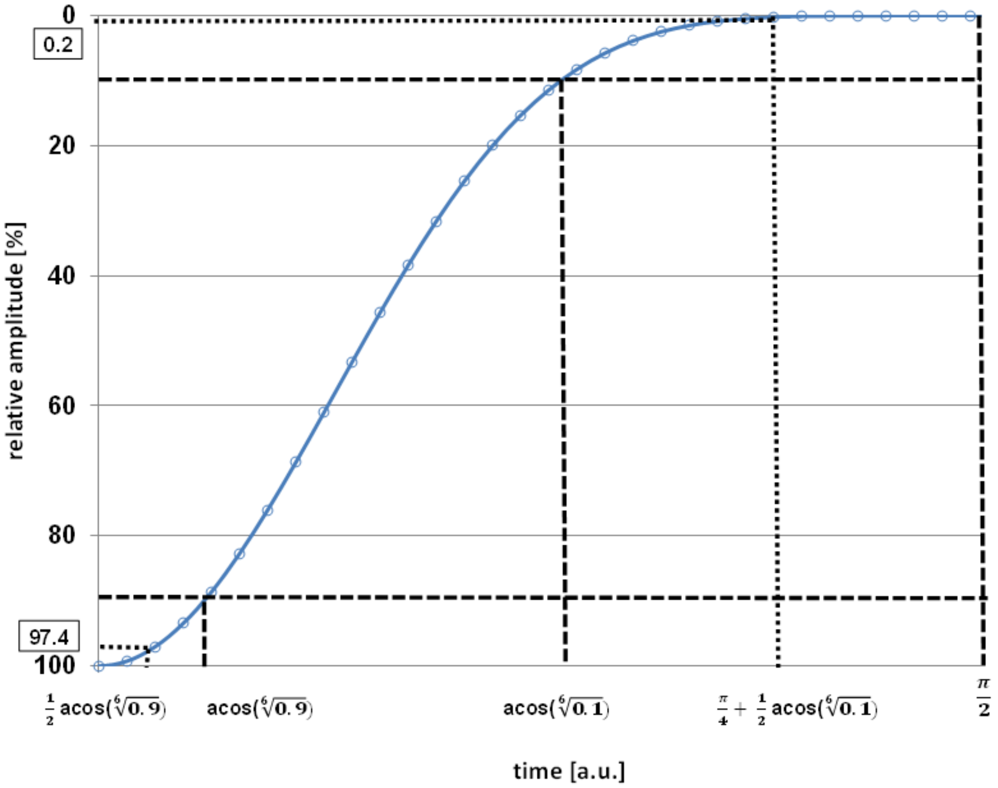

Figure S3: *The estimated error of a regular* $\cos^6$ *waveform. The dotted lines depict the median position for the end-inhale and end-exhale bins, which each have a bin size of 10% of the amplitude.*

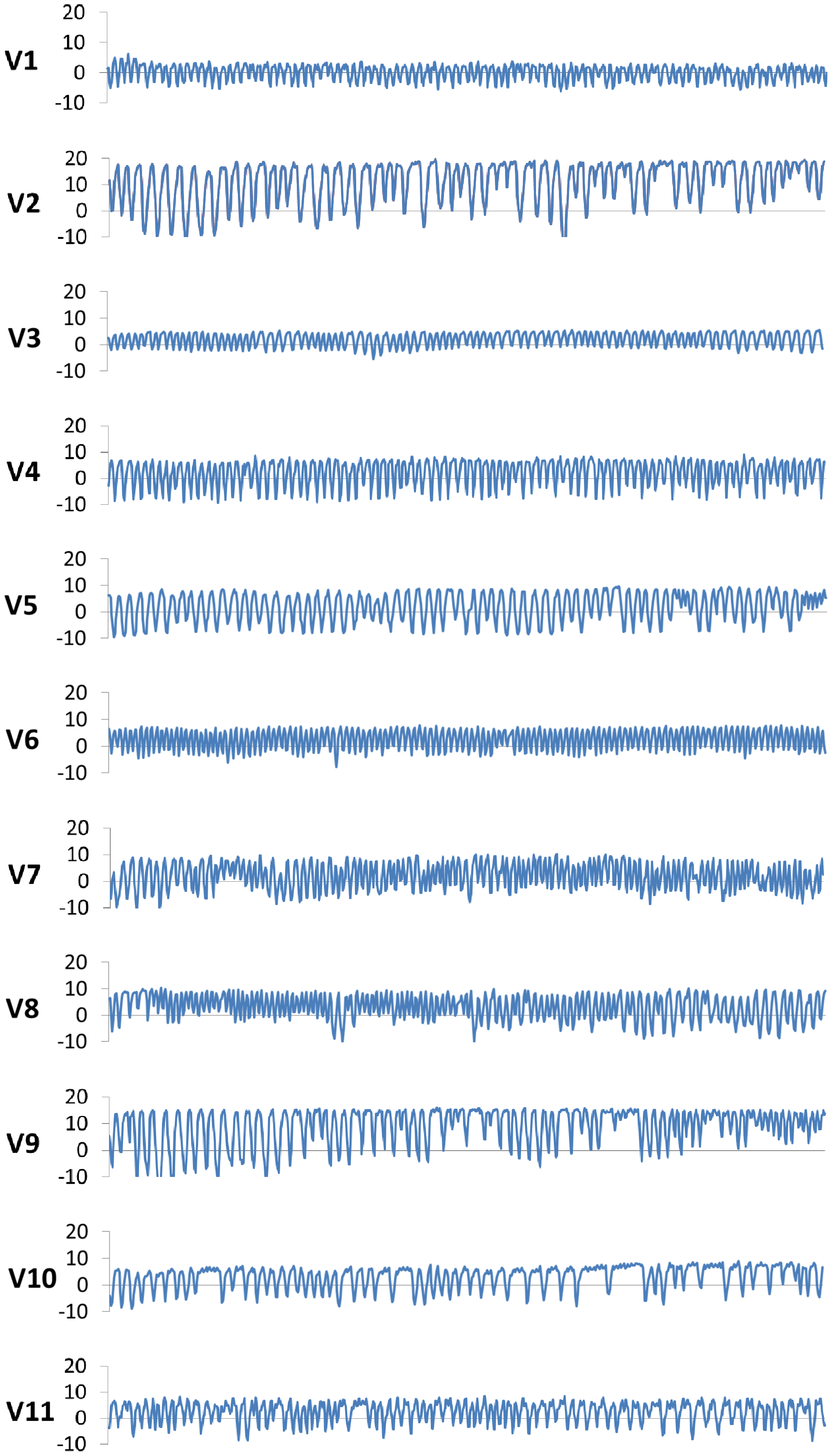

V1
V2
V3
V4
V5
V6
V7
V8
V9
V10
V11
20
10
0
-10

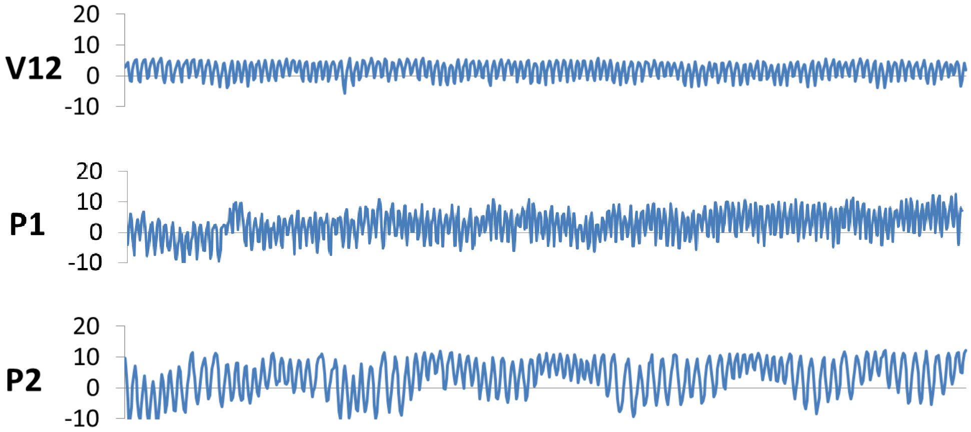

Figure S4: *Respiratory signals over 6 minutes of included subjects: healthy volunteers V1-12 and patients P1,P2. Y-axis denotes diaphragm position in mm, X-axis denotes time. Various breathing irregularities were present in both patients and volunteers, characterized by differences in amplitudes, drifts, hiccups, and large variation in end-inhalation diaphragm positions.*

Table S1: *Quality parameters values for each subject for 4DMRIs reconstructed with four outlier rejection and binning strategies.*

Subject	Min95					Phase					MaxIE					MeanIE				
	DI [%]	RC [%]	IBV [mm]	IR [mm]	S	DI [%]	RC [%]	IBV [mm]	IR [mm]	S	DI [%]	RC [%]	IBV [mm]	IR [mm]	S	DI [%]	RC [%]	IBV [mm]	IR [mm]	S
V1	95.0	99.1	1.0	7.9	0.98	100.0	100.0	2.2	9.9	0.94	100.0	91.8	0.9	9.9	0.89	81.0	96.4	1.0	6.5	0.96
V2	95.0	93.6	2.4	23.5	0.92	100.0	100.0	12.2	39.7	0.50	100.0	78.2	3.5	39.7	*	75.0	93.6	1.9	18.9	0.84
V3	95.0	99.1	0.8	7.5	1.00	100.0	99.1	3.8	10.9	0.97	100.0	80.0	1.1	10.9	0.80	77.0	97.3	0.6	6.4	1.00
V4	95.0	94.6	1.7	15.6	0.89	100.0	100.0	6.5	18.4	0.90	100.0	90.0	2.0	18.4	0.89	79.0	94.6	1.5	13.1	0.89
V5	95.0	98.2	1.6	16.7	0.82	100.0	100.0	6.7	18.6	0.61	100.0	98.2	1.9	18.6	0.80	76.0	91.8	1.5	13.7	0.75
V6	95.0	98.2	1.2	10.7	0.84	100.0	100.0	5.6	15.7	0.79	100.0	80.0	1.6	15.7	0.69	78.0	97.3	1.0	9.1	0.85
V7	95.0	97.3	1.7	15.7	0.96	100.0	97.3	8.9	20.6	0.28	100.0	90.0	2.1	20.6	0.85	74.0	99.1	1.4	11.7	0.97
V8	95.0	99.1	1.5	15.5	0.54	100.0	100.0	4.0	20.5	0.80	100.0	90.9	1.5	20.5	0.45	73.0	96.4	1.7	10.7	0.57
V9	95.0	92.7	1.9	20.9	0.96	100.0	100.0	10.3	32.1	0.72	100.0	83.6	2.4	32.1	0.67	77.0	94.6	1.5	15.9	0.97
V10	95.0	99.1	1.0	13.5	0.98	100.0	97.3	5.5	17.0	0.93	100.0	91.8	1.3	17.0	0.89	75.0	97.3	1.0	11.3	0.98
V11	95.0	94.6	1.4	12.9	0.91	100.0	100.0	6.4	17.2	0.64	100.0	83.6	1.6	17.2	0.64	84.0	95.5	1.2	10.6	0.91
V12	95.0	96.4	1.0	8.3	0.98	100.0	100.0	4.1	11.7	0.88	100.0	80.0	1.2	11.7	0.79	80.0	97.3	0.9	6.4	0.97
P1	95.0	96.4	2.5	15.9	0.88	100.0	100.0	5.0	25.3	0.74	100.0	83.6	2.5	25.3	0.77	73.0	98.2	2.3	10.5	0.94
P2	95.0	79.2	2.6	26.5	0.87	100.0	99.2	6.4	40.0	0.95	100.0	76.2	3.0	40.0	0.78	31.0	69.2	2.9	17.7	0.58
Mean	95.0	95.5	1.6	15.1	0.90	100.0	99.5	6.2	21.3	0.76	100.0	85.6	1.9	21.3	0.76	73.8	94.2	1.5	11.6	0.87
SD	0.0	5.2	0.6	5.7	0.12	0.0	1.0	2.7	9.8	0.20	0.0	6.5	0.8	9.8	0.12	12.7	7.4	0.6	4.0	0.14

*For volunteer 2 (V2), S could not be determined as fitting failed because of missing images (low RC) in consecutive slices. DI = data included, RC = reconstruction completeness, IBV = intra-bin variation, IR = inclusion range, S = image smoothness
